# Semaglutide Concurrently Improves Vascular and Liver Indices in Patients With Type 2 Diabetes and Fatty Liver Disease

**DOI:** 10.1210/jendso/bvae122

**Published:** 2024-07-08

**Authors:** Emmanouil Korakas, Aikaterini Kountouri, George Pavlidis, Evangelos Oikonomou, Emmanouil Vrentzos, Eleni Michalopoulou, Vasiliki Tsigkou, Konstantinos Katogiannis, Loukia Pliouta, Konstantinos Balampanis, Sotirios Pililis, Konstantinos Malandris, Apostolos Tsapas, Gerasimos Siasos, Ignatios Ikonomidis, Vaia Lambadiari

**Affiliations:** 2nd Department of Internal Medicine Research Unit and Diabetes Centre Attikon Hospital, Medical School National and Kapodistrian University of Athens, 12462 Athens, Greece; 2nd Department of Internal Medicine Research Unit and Diabetes Centre Attikon Hospital, Medical School National and Kapodistrian University of Athens, 12462 Athens, Greece; 2nd Department of Cardiology Laboratory of Preventive Cardiology and Echocardiography Department Attikon Hospital, Medical School National and Kapodistrian University of Athens, 12462 Athens, Greece; 3rd Department of Cardiology, National and Kapodistrian University of Athens, Medical School, Sotiria Chest Disease Hospital, 11527 Athens, Greece; Rheumatology and Clinical Immunology Unit, 4th Department of Internal Medicine, Attikon University Hospital, Joint Rheumatology Program, National and Kapodistrian University of Athens Medical School, 12462 Athens, Greece; 2nd Department of Cardiology Laboratory of Preventive Cardiology and Echocardiography Department Attikon Hospital, Medical School National and Kapodistrian University of Athens, 12462 Athens, Greece; 3rd Department of Cardiology, National and Kapodistrian University of Athens, Medical School, Sotiria Chest Disease Hospital, 11527 Athens, Greece; 2nd Department of Cardiology Laboratory of Preventive Cardiology and Echocardiography Department Attikon Hospital, Medical School National and Kapodistrian University of Athens, 12462 Athens, Greece; 2nd Department of Internal Medicine Research Unit and Diabetes Centre Attikon Hospital, Medical School National and Kapodistrian University of Athens, 12462 Athens, Greece; 2nd Department of Internal Medicine Research Unit and Diabetes Centre Attikon Hospital, Medical School National and Kapodistrian University of Athens, 12462 Athens, Greece; 2nd Department of Internal Medicine Research Unit and Diabetes Centre Attikon Hospital, Medical School National and Kapodistrian University of Athens, 12462 Athens, Greece; Clinical Research and Evidence-Based Medicine Unit, Aristotle University of Thessaloniki, 54124 Thessaloniki, Greece; Clinical Research and Evidence-Based Medicine Unit, Aristotle University of Thessaloniki, 54124 Thessaloniki, Greece; 3rd Department of Cardiology, National and Kapodistrian University of Athens, Medical School, Sotiria Chest Disease Hospital, 11527 Athens, Greece; 2nd Department of Cardiology Laboratory of Preventive Cardiology and Echocardiography Department Attikon Hospital, Medical School National and Kapodistrian University of Athens, 12462 Athens, Greece; 2nd Department of Internal Medicine Research Unit and Diabetes Centre Attikon Hospital, Medical School National and Kapodistrian University of Athens, 12462 Athens, Greece

**Keywords:** semaglutide, nonalcoholic fatty liver disease, arterial stiffness, glycocalyx, fibrosis

## Abstract

**Context:**

The cardiovascular benefits of semaglutide are established; however, its effects on surrogate vascular markers and liver function are not known.

**Objective:**

To investigate the effects of semaglutide on vascular, endothelial, and liver function in patients with type 2 diabetes (T2DM) and nonalcoholic fatty liver disease (NAFLD).

**Methods:**

Overall, 75 consecutive subjects with T2DM and NAFLD were enrolled: 50 patients received semaglutide 1 mg (treatment group) and 25 patients received dipeptidyl peptidase 4 inhibitors (control group). All patients underwent a clinical, vascular, and hepatic examination with Fibroscan elastography at 4 and 12 months after inclusion in the study.

**Results:**

Treatment with semaglutide resulted in a reduction of Controlled Attenuation Parameter (CAP) score, E fibrosis score, NAFLD fibrosis score, Fibrosis-4 (FIB-4) score and perfused boundary region (PBR) at 4 and at 12 months (*P* < .05), contrary to controls. Patients treated with semaglutide showed a greater decrease of central systolic blood pressure (SBP) (−6% vs −4%, *P* = .048 and −11% vs −9%, *P* = .039), augmentation index (AIx) (−59% vs −52%, *P* = .041 and −70% vs −57%, *P* = .022), and pulse wave velocity (PWV) (−6% vs −3.5%, *P* = .019 and −12% vs −10%, *P* = .036) at 4 and at 12 months, respectively. In all patients, ΔPWV and ΔPBR were correlated with a corresponding reduction of CAP, E fibrosis, NAFLD fibrosis, and FIB-4 scores.

**Conclusion:**

Twelve-month treatment with semaglutide simultaneously improves arterial stiffness, endothelial function, and liver steatosis and fibrosis in patients with T2DM and NAFLD.

Nonalcoholic fatty liver disease (NAFLD) is now considered the most frequent liver disease worldwide, with its global prevalence reaching up to 32.4% [1]. It is characterized by the accumulation of fat in the liver, which, if untreated, can progress to nonalcoholic steatohepatitis (NASH), fibrosis, and cirrhosis [2]. Its close association and the common pathogenetic pathways with elements of metabolic syndrome, such as insulin resistance, type 2 diabetes mellitus (T2DM), and dyslipidemia, have been well-established in recent years. More specifically, insulin resistance promotes enhanced hepatic gluconeogenesis and lipogenesis, while at the same time, it reduces muscle glucose uptake adipose tissue lipolysis. The cumulative effect is the increase in glucose and free fatty acid levels, which, in turn, are stored as triglycerides in the liver [3]. Current evidence shows that women with prediabetes and T2DM have higher risk for NAFLD than men [4]. Despite some initially promising effects of pioglitazone and vitamin E on liver function, there is currently no officially approved treatment for NAFLD, with the only effective measure being the weight loss of at least 10%. Due to their beneficial effects on glycemic control and obesity, glucagon-like peptide-1 receptor agonists (GLP-1RAs) pose as a potential therapeutic option for NAFLD, and growing evidence is building on their efficacy, through mechanisms which extend beyond weight loss [5].

Apart from their metabolic actions, GLP-1RAs exert substantial cardiovascular benefits. In 5 of 7 clinical trials with cardiovascular outcome, statistically significant reductions in major cardiovascular adverse events (MACE) were reported, and these effects were independent of their glucose-lowering actions [6]. The most prominent mechanism seems to be the amelioration of atherosclerosis. However, due to the detrimental effects of diabetes on the endothelium early in the course of the disease, endothelial dysfunction has been considered a reliable predictor of cardiovascular disease in recent years [7]. Apart from its barrier function and regulation of cell permeability, the endothelium participates in the regulation of blood pressure, immune and inflammatory cascades, and coagulation. T2DM and obesity promote endothelial dysfunction mainly through a reduction in nitric oxide (NO) bioavailability, but also through the accumulation of proinflammatory cytokines and adhesion molecules. Arterial stiffness, as assessed by pulse wave velocity (PWV), has also been found to be elevated in T2DM [8]. The endothelium may present a direct target for GLP-1RAs, as it is suggested by the presence of the corresponding receptor in endothelial cells and macrophages, and by several in vitro studies where a protective role of GLP-1RA treatment has been demonstrated [9]. A credible estimation of endothelial integrity can be achieved through the imaging of the endothelial glycocalyx. This mesh of glycoproteins, proteoglycans, and associated glycosaminoglycans covers the endothelium and is severely affected in states of hyperglycemia, without any established treatment at the time being. Several clinical studies which have investigated changes in vascular indices with GLP-1RAs have been published in recent years; however, data is still scarce [10-13].

Semaglutide, a long-acting GLP-1RA, presents the most beneficial metabolic actions and has been under the spotlight for its possible role in NAFLD [14, 15]. On the other hand, despite its impressive cardiovascular effects, no sufficient data regarding surrogate markers of vascular function exists. The aim of this study was to examine the changes in markers of arterial stiffness, endothelial function, liver steatosis, and fibrosis in subjects with T2DM and NAFLD after 12 months of treatment with semaglutide.

## Materials and Methods

### Study Design

Overall, 75 consecutive subjects with T2DM and NAFLD, who attended the Diabetes Outpatients Clinic were enrolled in the study. Of these subjects, 50 patients received semaglutide 1 mg (treatment group) and 25 patients (control group) received dipeptidyl peptidase 4 (DPP-4) inhibitors (either linagliptin or alogliptin or vildagliptin or sitagliptin). All patients underwent a clinical, vascular, and hepatic examination with Fibroscan elastography at 4 and 12 months after inclusion in the study. Exclusion criteria were malignancies, chronic inflammatory disease, chronic kidney disease (estimated glomerular filtration rate < 60 mL/min/m^2^ for a period of at least 90 days), peripheral vascular disease, retinopathy, alcohol consumption of > 2 drinks per day for men and > 1 drink per day for women, and previous therapy with a glucagon-like peptide-1 (GLP-1) agonist. None of the female patients was on hormone replacement treatment. No specific dietary recommendations other than the standard ones for T2DM were given. The investigation conforms to the principles outlined in the Declaration of Helsinki. The study protocol was approved by the University General Hospital “Attikon” Institutional Review Board. In addition, all participants gave their written informed consent.

### Endothelial Glycocalyx

The perfused boundary region (PBR) of the sublingual arterial microvessels with a diameter that ranged from 5 to 25 μm was measured using Sidestream Dark Field imaging (Microscan, Glycocheck, Microvascular Health Solutions Inc, Salt Lake City, UT). The PBR is the cell-poor layer that results from the separation between the flowing red blood cell column and plasma on the surface of the vascular lumen. An increased PBR value indicates a deeper penetration of blood cells into the luminal part of the glycocalyx and is, thus, an index of damaged glycocalyx integrity.

### Central Hemodynamics

We measured the carotid-femoral pulse wave velocity (PWV), augmentation index (AIx), and central aortic pressures (central systolic and diastolic) using tonometry by Complior (Alam Medical, Vincennes, France). Normal values were PWV < 10 m/s. AIx was calculated using the formula: 100 × (P2 − P1)/PP, where P2 is the late backward systolic wave, P1 is the early forward systolic wave, and PP is the pulse pressure.

### Liver Testing

Liver steatosis and stiffness was measured using Fibroscan® Mini + 430 (Echosens, Paris, Île-de-France). Controlled Attenuation Parameter (CAP) score was used as an index of liver fat content, with normal values being < 238 dB/m. The E score was used as an index of liver fibrosis, with normal values being 2 to 6 kPa.

### Statistical Analysis

Statistical analysis was conducted using the Statistical Package for Social Sciences (IBM SPSS Statistics for Windows, Version 26.0. Armonk, NY, USA). Continuous variables were expressed as mean ± SD with the exception of AIx values, which were presented as median with interquartile range (first quartile—third quartile) according to their distribution. Differences in continuous variables were evaluated by the Student *t* test and Mann-Whitney U test, as appropriate. Categorical variables were presented as numbers with corresponding percentages and were analyzed performing chi-square test or Fisher exact test. All analyses were intention to treat. Analysis of variance (ANOVA) for repeated measurements was performed for (a) measurements of the examined markers at baseline, and at 4 and 12 months of antidiabetic treatment, which was considered as a within-subject factor and (b) for the effects of different treatments, as a between-subject factor (semaglutide and control). Age, sex, hypertension, hyperlipidemia, concomitant medical treatments, fasting glucose, glycated hemoglobin (HbA1c), body mass index (BMI), mean blood pressure and their changes at 4 and 12 months of treatment were included as covariates in the model. The F and *P* values of the interaction between time of measurement of the examined markers and the type of treatment were calculated. Moreover, the F and the corresponding *P* values of the comparison between treatments were estimated. All statistical tests were two-tailed and a *P* < .05 was considered statistically significant.

## Results

### Baseline Characteristics

The baseline characteristics of the study population are presented in Table 1. The mean age of the participants was 57 ± 12 years and 61% of them were male. Additionally, 26% of the patients had overt coronary artery disease (CAD). All patients had similar age, sex, BMI, risk factors, and cardiovascular medication usage. Furthermore, there were no differences in baseline fasting glucose levels and HbA1c between the 2 study groups (*P* > .05; Table 2).

**Table 1.** Demographic and clinical characteristics of the study population

	Total (n = 75)	Semaglutide (n = 50)	Controls (n = 25)	*P* value
Age, years	57 ± 12	57.5 ± 11	56 ± 13	.643
Sex, male, n (%)	46 (61)	31 (62)	15 (60)	.902
CAD, n (%)	20 (26)	14 (28)	6 (24)	.211
Risk factors, n (%)				
Hypertension	47 (62)	33 (66)	14 (56)	.197
Hyperlipidemia	42 (56)	29 (58)	13 (52)	.516
Current smoker	41 (54)	28 (56)	13 (52)	.334
Family history CAD	21 (28)	14 (28)	7 (28)	.979
Medications, n (%)				
ACEi/ARBs	20 (26)	14 (28)	6 (24)	.858
CCB	18 (24)	13 (26)	5 (20)	.258
β-Blockers	17 (22)	10 (20)	7 (28)	.176
Diuretics	19 (25)	13 (26)	6 (24)	.649
MRA	7 (9)	5 (10)	2 (8)	.875
Statins	39 (52)	28 (56)	11 (44)	.292
Fibrates	10 (13)	6 (12)	4 (16)	.488
Antiplatelets	20 (26)	14 (28)	6 (24)	.211

Data are expressed as the mean ± SD or number (%).

Abbreviations: ACEi, angiotensin-converting enzyme inhibitors; ARBs, angiotensin receptor blockers; CAD, coronary artery disease; CCB, calcium channel blockers; MRA, mineralocorticoid receptor antagonists.

**Table 2.** Effects of semaglutide vs control on glycemic control, endothelial and vascular markers, and liver steatosis and fibrosis during the study period

	Total (n = 75)	Semaglutide (n = 50)	Controls (n = 25)
Fasting glucose (mg/dL)			
Baseline	152 ± 47	155 ± 46	148 ± 49
4 months	125 ± 25*^a^*	127 ± 26*^b^*	122 ± 25*^c^*
12 months	112 ± 19*^b^*	110 ± 20*^b^*	119 ± 13*^c^*
HbA1c (%)			
Baseline	7.42 ± 0.94	7.51 ± 0.95	7.27 ± 0.91
4 months	6.48 ± 0.76*^a^*	6.45 ± 0.73*^a^*	6.55 ± 0.84*^c^*
12 months	6.07 ± 0.47*^b^*	6.06 ± 0.58*^b^*	6.15 ± 0.49*^c^*
BMI (kg/m^2^)			
Baseline	34 ± 6.4	35 ± 5.8	31.8 ± 7.4
4 months	31.7 ± 5.8*^a^*	32.2 ± 5.5*^a^*	30.4 ± 6.4*^b^*
12 months	29.4 ± 3.6*^b^*	30.4 ± 3.5*^b^*	29.3 ± 4.5*^b,d^*
CAP score (dB/m)			
Baseline	312 ± 40	319 ± 37	298 ± 45
4 months	284 ± 50*^a^*	283 ± 46*^a^*	286 ± 59
12 months	264 ± 39*^b^*	270 ± 40*^c^*	279 ± 38*^d^*
E (kPa)			
Baseline	6.6 ± 3.7	6.9 ± 3.9	6.1 ± 4.4
4 months	5.4 ± 2.1*^c^*	5.5 ± 2.2*^c^*	5.4 ± 2.1
12 months	5.1 ± 1.7*^c^*	5.2 ± 1.5*^c^*	5 ± 1.8*^d^*
NAFLD fibrosis Score			
Baseline	0.15 ± 1.19	0.058 ± 1.1	0.45 ± 1.39
4 months	−0.058 ± 1.06*^b^*	−0.156 ± 1.04*^b^*	0.25 ± 1.21
12 months	0.12 ± 0.63*^c^*	−0.020 ± 0.53*^b^*	0.26 ± 0.96*^d^*
FIB-4 score			
Baseline	1.14 ± 0.52	1.12 ± 0.46	1.21 ± 0.72
4 months	1.03 ± 0.55*^a^*	1 ± 0.41*^b^*	1.14 ± 0.52
12 months	1.06 ± 0.51*^b^*	1.01 ± 0.29*^c^*	1.17 ± 0.67*^d^*
PBR 5-25 μm			
Baseline	2.25 ± 0.22	2.23 ± 0.23	2.29 ± 0.18
4 months	2.21 ± 0.25	2.21 ± 0.25	2.22 ± 0.24
12 months	2.03 ± 0.43*^c^*	1.99 ± 0.46*^c^*	2.19 ± 0.27*^d^*
SBP (mmHg)			
Baseline	142 ± 16	143 ± 15	140 ± 18
4 months	139 ± 18	141 ± 20	137 ± 15
12 months	133 ± 11*^c^*	135 ± 10*^c^*	132 ± 8*^c^*
DBP (mmHg)			
Baseline	84 ± 9	85 ± 8	82 ± 10
4 months	84 ± 9	84 ± 9	81 ± 11
12 months	83 ± 8	82 ± 12	79 ± 6
Central SBP (mmHg)			
Baseline	135 ± 19	136 ± 20	133 ± 19
4 months	128 ± 16*^c^*	128 ± 16*^c^*	127 ± 17*^c^*
12 months	121 ± 8*^c^*	121 ± 9*^c^*	120 ± 12*^c,d^*
AIx (%)			
Baseline	18.9 (4.6–28.4)	19.1 (4.8–28.6)	17 (2.8–25.4)
4 months	7.8 (2.1–15.4)*^c^*	7.8 (1.8–18.6)*^c^*	7.3 (2.7–15.5)*^c^*
12 months	6.1 (−6.2–18.1)*^c^*	5.7 (−6.5–12.3)*^c^*	8.1 (0.8–18.7)*^c,d^*
PWV (m/s)			
Baseline	11.5 ± 2.7	11.5 ± 2.6	11.4 ± 3
4 months	10.9 ± 2.5*^c^*	10.8 ± 2.2*^c^*	11 ± 3.2*^c^*
12 months	10.1 ± 1.5*^b^*	10.1 ± 1.7*^c^*	10.2 ± 1.1*^c,d^*

Data are presented as mean ± SD. Augmentation index (AIx) values are presented as median (first quartile—third quartile).

Abbreviations: BMI, body mass index; CAP, Controlled Attenuation Parameter; DBP, diastolic blood pressure; E, fibrosis score; HbA1c, glycosylated hemoglobin; NAFLD, nonalcoholic fatty liver disease; PBR, perfused boundary region; PWV, pulse wave velocity; SBP, systolic blood pressure.

^*a*^ *P* < .001 for comparisons of 4 or 12 months vs baseline.

^*b*^ *P* < .01.

^*c*^ *P* < .05.

^*d*^ *P* < .05 for time × treatment interaction obtained by repeated measures ANOVA.

### Effect of Treatment on Glycemic Control and Weight Loss

All patients had improved fasting glucose levels and HbA1c at 4 months (*P* < .001) and at 12 months (*P* = .002 and *P* = .001, respectively). Furthermore, BMI was reduced in the whole study population at 4 months (*P* = .002) and at 12 months post treatment (*P* = .001). However, there was a significant interaction of time with the type of antidiabetic treatment and BMI (F = 4.292, *P* for interaction = .021). Patients receiving semaglutide showed a greater decrease of BMI at 4 months (−8% vs −4%, *P* = .025) and at 12 months (−13% vs −7%, *P* = .008) compared with the control group.

### Effect of Treatment on Liver Steatosis and Fibrosis

Compared to baseline, all patients had significantly reduced CAP score, E fibrosis score, NAFLD score, and Fibrosis-4 (FIB-4) score at 4 and 12 months (*P* < .05 for all comparisons; Table 2). Of note, a significant interaction of follow-up time with the type of treatment was observed regarding CAP score (F = 2.982, *P* for interaction = .042), E fibrosis score (F = 4.542, *P* for interaction = .039) NAFLD score (F = 6.348, *P* for interaction = .038), and FIB-4 score (F = 3.035, *P* = .032) in a model including age, sex, hypertension, hyperlipidemia, concomitant medical treatments, fasting glucose, HbA1c, BMI, mean blood pressure, and their changes at 4 and 12 months of treatment. Treatment with semaglutide resulted in a reduction of CAP score, E fibrosis score, NAFLD score, and FIB-4 score at 4 and at 12 months (*P* < .05 for all comparisons), whereas controls presented no significant reduction of the aforementioned markers at the same time points (*P* > .05 for all comparisons; Table 2).

### Effect of Treatment on Endothelial and Vascular Markers

All patients had improved endothelial glycocalyx integrity, as assessed by the reduced PBR at 12 months of treatment (*P* = .037). Nevertheless, there was a significant interaction of time with the type of treatment and PBR (F = 3.129, *P* for interaction = .040). Semaglutide led to a remarkable decrease of PBR at 12 months (*P* = .026), whereas the control group showed no significant reduction of PBR at the same time (*P* = .482). Moreover, all patients had reduced SBP at 12 months and decreased central SBP, AIx, and PWV at 4 and at 12 months of treatment (*P* < .05; Table 2). Interestingly, a significant interaction of follow-up time with the type of treatment according to central SBP (F = 4.238, *P* for interaction = .041), AIx (F = 3.228, *P* = .039), and PWV (F = 3.743, *P* for interaction = .032). More specifically, patients on semaglutide showed a greater decrease of central SBP (−6% vs −4%, *P* = .048 and −11% vs −9%, *P* = .039), AIx (−59% vs −52%, *P* = .041 and −70% vs −57%, *P* = .022), and PWV (−6% vs −3.5%, *P* = .019 and −12% vs −10%, *P* = .036) at 4 and at 12 months, respectively (Table 2).

### Sex-Related Differences in the Effect of Treatment on Weight Loss, Liver Steatosis, and Vascular Markers

Among patients who were treated with semaglutide, women displayed a greater BMI reduction compared to men at 4 and 12 months of treatment (−10.2% vs −6.7%, *P* = .039 and −15.8% vs −11.3%, *P* = .014, respectively) in parallel with a greater reduction of CAP score (−13.7% vs −9.5%, *P* = .044 and −17.6% vs −13.9%, *P* = .018), FIB-4 score (−12.1% vs −9.8%, *P* = .045 and −11% vs −9%, *P* = .031), and central SBP (−7.2% vs −5.3%, *P* = .042 and −12.4% vs −10.2%, *P* = .039) at the same time points. There were no statistically significant sex-related differences in the aforementioned markers in the placebo group (*P* > .05, data not shown).

### Correlation of Metabolic, Endothelial, and Vascular Markers With Liver Steatosis and Fibrosis

In all study patients, baseline HbA1c was correlated with CAP score (*r* = 0.34, *P* = .018), E fibrosis score (*r* = 0.32, *P* = .027), PWV (*r* = 0.39, *P* = .004), and PBR (*r* = 0.28, *P* = .045). In addition, PBR was associated with CAP score (*r* = 0.26, *P* = 0.044), E fibrosis score (*r* = 0.25, *P* = .048), and central SBP (*r* = 0.29, *P* = .036).

In the whole study population, the percentage reduction of arterial stiffness, as assessed by PWV, was correlated with a corresponding reduction of liver steatosis markers, namely CAP score (*r* = 0.487, *P* = .030 and *r* = 0.529, *P* = .028), E fibrosis score (*r* = 0.449, *P* = .008 and *r* = 0.565, *P* = .007), NAFLD fibrosis score (*r* = 0.253, *P* = .039 and *r* = 0.548, *P* = .001), and FIB-4 (*r* = 0.281, *P* = .034 and *r* = 0.625, *P* = .002), at 4 and 12 months after treatment, respectively.

After 12-month treatment with semaglutide, the percentage decrease of PBR was associated with the percentage reduction of CAP (*r* = 0.323, *P* = .035), E fibrosis score (*r* = 0.342, *P* = .023), NAFLD fibrosis score (*r* = 0.486, *P* = .009), and FIB-4 (*r* = 0.317, *P* = .041).

## Discussion

In this study, we showed that treatment with semaglutide for 12 months showed statistically significant reductions in BMI, PWV, and central SBP, and improved endothelial glycocalyx thickness. In addition, it significantly improved CAP score and liver stiffness, and a positive interrelation between these indices and improvements in vascular function was noticed.

To date, there are few studies which have investigated the effect of GLP-1RAs on arterial stiffness and endothelial function, and the majority of them have included liraglutide as an interventional drug. In a study by Lambadiari et al [11], 6-month treatment with liraglutide reduced PWV (11.8 ± 2.5 vs 10.3 ± 3.3 m/s) in recipients, compared to subjects who received only metformin, and this positive effect was reproduced in another report by the same group, where liraglutide resulted in a statistically significant reduction in PWV, which was sustained after 12 months of treatment (11.6 ± 2.8 vs 10.5 ± 1.9 m/s) [12]. In addition, liraglutide treatment alone improved endothelial glycocalyx integrity, reducing PBR by 2.9% after 12 months (2.1 ± 0.29 vs 2.04 ± 0.2 μm). In a meta-analysis of 26 studies, which included patients on newer antidiabetic drugs, GLP-1RAs significantly reduced PWV (pooled mean difference [MD] = −1.97; 95% CI, −2.65 to −1.30; *P* < .001), but, surprisingly, did not present the same beneficial effect on flow-mediated dilation of the brachial artery (FMD), which is an index of endothelial function (pooled MD = 2.37%; 95% CI, −0.51 to 5.25; *P* = .107) [16]. On the contrary, apart from reductions in PWV (11.2 ± 0.9 vs 10.6 ± 0.8 m/s), 9-month treatment with dulaglutide improved endothelial function as well, as described by an increase in the reactive hyperemia index [13]. However, there is a serious paucity of evidence regarding semaglutide, and most data derive from animal or in vitro studies. In endothelial progenitor cells, semaglutide inhibited lipopolysaccharide-induced macrophage expression of miR-155 in exosomes and improved cell viability and inflammatory status [17]. In ApoE-/- mice following vascular injury and blood flow perturbation, semaglutide reduced the intimal and medial area by ∼66% and ∼11%, respectively and reduced inflammatory markers [18], and this anti-inflammatory, antiproliferative effect on vascular remodeling has been observed with GLP-1RAs in previous in vitro studies with vascular smooth muscle cells [19, 20]. An improvement in aortic injury was also detected in obese C57BL/6J mice, semaglutide reduced PWV, and histology analysis showed decreased collagen levels and attenuated elastin damage [21]. Clinical data with semaglutide, however, are scarce, and this is considered a novelty of our study. A direct comparison with our study can be made only with the work by Navodnik et al [22], who enrolled patients with type 1 diabetes mellitus and measured FMD and forearm blood flow as parameters of endothelial function, as well as PWV and peripheral resistance as parameters of arterial stiffness. A statistically significant 1.9-fold improvement in FMD was noted in patients who received semaglutide for 3 months, along with a decrease in peripheral resistance, while there was a favorable tendency regarding PWV (7.32 ± 1.66 m/s vs 6.98 ± 2.0 m/s), and AIx (29.9 ± 9.1 vs 27.4 ± 10.2), albeit it was not statistically significant. Even more importantly, these effects were independent of the improvements in BMI, HbA1c, and glucose variability, a fact that confirms that semaglutide exerts glucose-independent actions on the vascular wall. Of course, the impact of insulin resistance and obesity that accompany T2DM on vascular function cannot be ignored, and this should be taken into account when comparing these results to our study, which, to our knowledge, is the only one to directly measure the effects of semaglutide on arterial stiffness and endothelial glycocalyx in subjects with T2DM.

The mechanisms through which GLP-1RAs exert their vasculoprotective actions have not yet been fully clarified, but they certainly extend beyond metabolic improvement. The presence of GLP-1 receptors (GLP-1R) in endothelial cells is a matter of debate [23]. GLP-1R expression has been detected in mouse mesenteric or coronary arteries in immunohistochemistry studies utilizing GLP-1R antibodies; however, their validity has been proven to be nonspecific in some cases [24]. On the other hand, in human models, Western blotting has confirmed GLP-1R protein expression in human umbilical vein endothelial cells (HUVECs) [25], but in human heart samples, full-length GLP1-R mRNA transcripts have not been detected [26]. Despite these contradictory results, the direct actions of GLP-1RAs on endothelial cells cannot be disputed. Treatment with liraglutide or exendin-4 increased endothelial NO synthase (eNOS) phosphorylation and NO production in a 5′AMP-activated protein kinase (AMPK)-dependent manner [27, 28]. In addition, liraglutide reduced tumor necrosis factor alpha (TNFα)-induced nuclear factor kappa B activation in HUVECs, along with the expression of adhesion molecules, such as VCAM-1 and MCP-1 [29]. In a previous study by our group, 6-month treatment with liraglutide reduced malondialdehyde and protein carbonyls, which are markers of oxidative stress; similar effects have been reproduced in other studies [11, 30]. In general, whether the vasculoprotective action of GLP1-RAs is mainly attributed to local GLP-1R stimulation or the peripheral effects that improve atherosclerosis is yet to be elucidated [31, 32]; however, the anti-inflammatory properties of this drug class indisputably play a pivotal role.

Regarding the effect of semaglutide on NAFLD and NASH, the existing data support a significant improvement in the degree of steatosis, but less promising results in terms of fibrosis. In a double-blind, placebo-controlled phase 2 trial, in which adults with biopsy-confirmed NASH-related cirrhosis and BMI of 27 kg/m² were assigned to semaglutide 2.4 mg or placebo, no statistically significant difference between the 2 groups in the proportion of patients who had an improvement in liver fibrosis of 1 stage or more was noted after 48 weeks, and the same applied for the endpoint of NASH resolution [33]. On the contrary, however, semaglutide led to reductions in liver enzymes and liver steatosis, depicted by > 30% decrease in magnetic resonance imaging proton density fat fraction (MRI-PDFF). A very interesting study, which can be more directly compared to our results due to the use of Fibroscan, was a 72-week, double-blind phase 2 trial, involving patients with biopsy-confirmed NASH and liver fibrosis of stage F1, F2, or F3, where patients were assigned to once-daily semaglutide at a dose of 0.1, 0.2, or 0.4 mg or corresponding placebo [34]. Again, the improvement of 43% in fibrosis stage in the 0.4-mg group was not statistically significant compared to placebo; however, NASH resolution was observed in 59% of the patients in the 0.4-mg group, which was significant compared to 17% in the placebo group. The surprising discrepancy between fibrosis regression and NASH resolution is not completely understood; the short duration of the aforementioned trials has been proposed as a possible confounding factor. However, dose-dependent reductions in liver enzymes were observed, as was the case for liver stiffness assessed by Fibroscan similar to our study. Contrary to our results, Flint et al [35] did not observe any significant differences in liver stiffness either at week 24 or 48 of semaglutide treatment, although it should be noted that liver stiffness was assessed by magnetic resonance elastography (MRE) and not by Fibroscan, so the difference between the 2 studies could be attributed to methodological differences. More subjects achieved a ≥ 30% reduction in liver fat content at weeks 24, 48, and 72, (all *P* < .001) and decreases in liver enzymes with semaglutide, however, in agreement with our results. The poor results regarding liver stiffness could be partially explained by the low sensitivity of MRE to detect changes in less-advanced disease, again together with the short follow-up period of 48 weeks. In the study by Volpe et al [36], who enrolled a very similar cohort to ours, semaglutide 0.5 mg for 52 weeks led to at least 1-class reduction in the 4-point semiquantitative staging in NAFLD diagnosed via ultrasound, along with the expected improvements in liver enzymes, glycemic control, and visceral fat mass. In a systemic review and meta-analysis of 8 randomized controlled clinical trials involving 468 patients which evaluated the efficacy of GLP-1RAs in patients with T2DM and NAFLD, GLP-1RAs significantly decreased liver fat content measured using MRI, along with alanine aminotransferase (weighted mean difference [WMD] = −3.82 IU/L; 95% CI, −7.04 to −0.60; *P* = .02), aspartate aminotransferase (WMD = −2.4 IU/L; 95% CI, −4.55 to −0.25; *P* = .03), HbA1c, total cholesterol, and triglycerides, whereas no improvement was shown in FIB-4 index [37]. In general, most available data depend on studies involving liraglutide, and more studies with semaglutide are needed for robust associations to be shown. It should be noted that the use of various assessment methods of liver stiffness (Fibroscan, MRE, liver biopsy) is a very serious methodological difference among the various studies mentioned and, inevitably, it leads to serious difficulties in comparing both the populations studied and the eventual treatment effects.

The potential mechanisms of action of GLP-1RAs in NAFLD are various. Of course, as the mainstay of NAFLD treatment is weight loss, the beneficial effects of GLP-1RAs, especially liraglutide and semaglutide, are the most prominent treatment mechanism in these patients [38]. However, as insulin resistance is the cornerstone in the pathogenesis of both T2DM and NAFLD, pathways targeting hepatic and peripheral insulin resistance are of utmost importance. GLP-1 ligands are able to promote the phosphorylation of AKT, an effect that subsequently triggers the postreceptor signaling cascade downstream of insulin receptor substrate-2 (IRS-2), leading to reduced storage of hepatocyte triglycerides and, therefore, reduced hepatic steatosis [39]. GLP-1RAs also restore serum levels of adiponectin [40], inhibit de novo lipogenesis and peroxisome proliferator-activated receptor gamma (PPAR-γ) expression [41]. GLP-1R stimulation increases autophagy and mitigates endoplasmic reticulum (ER) stress and, therefore, it inhibits hepatocyte apoptosis and prevents further progression of liver damage [42]. The well-established reduction of chronic, low-grade inflammation and oxidative stress with GLP-1RAs also plays a decisive role [43, 44], and possibly explains the correlation between the enhancement in vascular indices and liver steatosis in our sample.

The association between PWV and CAP score is not newfound. In the study by Yu et al [45], it was found that significant fibrosis in NAFLD had an independent association with increased arterial stiffness after adjustment for cardiovascular risk factors, and the results in other studies were similar [46-48]. However, what needs to be pointed out is that, in the majority of data, the relationships between NAFLD and liver fibrosis with PWV were evident only in asymptomatic subjects, which is a remarkable difference compared to our results, where the participants had T2DM and, even more importantly, the CAP and E scores were associated with HbA1c. In addition, no data regarding the association of percentage change between liver and arterial markers exist, as the previous studies were mainly observational in nature. On the contrary, in our cohort, it was shown that there is a simultaneous improvement in PWV, PBR, and liver indices, which underlines the mutual pathophysiologic background of vascular and hepatic derangement, possibly in the form of chronic, low-grade inflammation.

In agreement with previous studies [49], in our study we found that women who received semaglutide presented a greater BMI reduction than men. The precise mechanism to explain sex-specific differences to weight loss caused by GLP-1RA remains unclear, but it could be related to increased drug exposure in women due to their lower average body weight. Moreover, the impact of sex hormones in the regulation of feeding behavior and the different rate of gastric emptying have been proposed for this difference between the sexes [50]. Interestingly enough, in the present study, a greater improvement of liver steatosis markers and central SBP was observed in woman compared to men. Therefore, semaglutide treatment may be useful to reduce the increased prevalence of NAFLD in diabetic women and to prevent cardiovascular complications.

A limitation of our trial is the low number of subjects with advanced disease, which may have made it difficult to detect a decrease in liver stiffness. In addition, the patients were not randomized to the study arms, but every effort was made to minimize any baseline differences and eliminate selection bias. On the other hand, the 12-month duration of our study is considered to be a sufficient follow-up duration, especially when compared with the existing literature. In addition, to our knowledge, it is the first study to examine the effects of semaglutide on endothelial glycocalyx, and even more importantly, its simultaneous effects on vascular disease and NAFLD.

In conclusion, 12-month treatment with semaglutide improves arterial stiffness, endothelial function, and liver steatosis and fibrosis in patients with T2DM and NAFLD. More large-scale, prospective clinical studies are needed to further establish these results and provide insights into the pathophysiological pathways through which semaglutide exerts its actions in the whole spectrum of cardiometabolic disease.

## Data Availability

The primary datasets generated and analyzed in the current study are not publicly available but are available from the corresponding author upon reasonable request.

## Clinical Trial Information

ClinicalTrials.gov registration ID: NCT06301191.

## Abbreviations

Aix: augmentation index
BMI: body mass index
FMD: flow-mediated dilation
GLP-1: glucagon-like peptide-1
GLP-1RA: glucagon-like peptide-1 receptor agonist
HbA1c: glycosylated hemoglobin
HUVEC: human umbilical vein endothelial cell
MRE: magnetic resonance elastography
MRI: magnetic resonance imaging
NAFLD: nonalcoholic fatty liver disease
NASH: nonalcoholic steatohepatitis
NO: nitric oxide
PBR: perfused boundary region
PWV: pulse wave velocity
SBP: systolic blood pressure
T2DM: type 2 diabetes mellitus
